# The Contribution of Bilingualism to Cognitive Functioning and Regional Brain Volume in Normal and Abnormal Aging

**DOI:** 10.1017/s1366728921000705

**Published:** 2021-11-09

**Authors:** Valeria L. Torres, Mónica Rosselli, David A. Loewenstein, Merike Lang, Idaly Vélez-Uribe, Fernanda Arruda, Joshua Conniff, Rosie E. Curiel, Maria T. Greig, Warren W. Barker, Miriam J. Rodriguez, Malek Adjouadi, David E. Vaillancourt, Russell Bauer, Ranjan Duara

**Affiliations:** 1 Department of Psychology, Florida Atlantic University, Davie, Florida, United States; 2 Florida Alzheimer’s Disease Research Center, Miami Beach and Gainesville, Florida, United States; 3 Department of Psychiatry and Behavioral Sciences and Center for Cognitive Neuroscience and Aging, Miller School of Medicine, University of Miami, Miami, Florida, United States; 4 Wien Center for Alzheimer’s Disease and Memory Disorders, Mount Sinai Medical Center, Miami Beach, Florida, United States; 5 Albizu University, Miami, Florida, United States; 6 Center for Advanced Technology and Education, College of Engineering, Florida International University, Miami, Florida, United States; 7 University of Florida Department of Applied Physiology and Kinesiology, Gainesville Florida, United States; 8 University of Florida College of Medicine, Department of Clinical and Health Psychology, Gainesville, Florida, United States

**Keywords:** Alzheimer’s disease, Mild Cognitive Impairment, bilingualism, entorhinal volume, hippocampal volume, brain biomarkers, aging

## Abstract

We examined the association between bilingualism, executive function (EF), and brain volume in older monolinguals and bilinguals who spoke English, Spanish, or both, and were cognitively normal (CN) or diagnosed with Mild Cognitive Impairment (MCI) or dementia. Gray matter volume (GMV) was higher in language and EF brain regions among bilinguals, but no differences were found in memory regions. Neuropsychological performance did not vary across language groups over time; however, bilinguals exhibited reduced Stroop interference and lower scores on Digit Span Backwards and category fluency. Higher scores on Digit Span Backwards were associated with a younger age of English acquisition, and a greater degree of balanced bilingualism was associated with lower scores in category fluency. The initial age of cognitive decline did not differ between language groups. The influence of bilingualism appears to be reflected in increased GMV in language and EF regions, and to a lesser degree, in EF.

## Introduction

One of the most significant controversies within the field of bilingualism research surrounds the idea that this dual-language capacity confers the speaker a cognitive advantage – namely, enhanced executive function (EF; Bialystok, Craik & Luk, 2008; Bialystok, Poarch, Luo & Craik, 2014b; Costa, Hernández & Sebastián-Gallés, 2008). EF is an umbrella term conceptualized to include different components of central cognitive control such as flexibility and inhibition (Baddeley & Hitch, 1974; Jurado & Rosselli, 2007; Lezak, 1983). It is theorized that the advantage of bilinguals in these domains results from the need to continually inhibit one language to manage linguistic interference while speaking the other language (Bialystok & Craik, 2010; Green, 1998). Another line of research portrays bilingualism as a contributor to cognitive reserve (Stern, 2009), thereby allowing bilinguals to maintain healthy cognitive function in aging, regardless of existing neuropathology, and delay the onset of cognitive impairment in aging (Perani, Farsad, Ballarini, Lubian, Malpetti, Fracchetti, Magnani, March & Abutalebi, 2017). However, not all research has found a bilingual advantage in the aging brain (Mungas, Early, Glymour, Zeki Al Hazzouri & Haan, 2018; Zahodne, Schofield, Farrell, Stern & Manly, 2014). This study aimed to explore this issue further.

### Bilingualism and executive function

The theory that bilingualism enhances EF originates from the idea that the habitual use of two languages requires the extensive and continual use of cognitive control mechanisms. A bilingual individual must rely on these abilities for effective communication. Engaging in constant cognitive control practice might enhance inhibitory and switching mechanisms (Green, 1998; Rosselli & Ardila, 2018). If these mechanisms are not language-specific, bilingualism should generate advantages in other specific cognitive domains related to inhibition and switching. These benefits have been described in tasks that require attentional control (Costa et al., 2008), inhibition (Bialystok et al., 2008), and spatial tasks of working memory (Luo, Craik, Moreno & Bialystok, 2013).

Despite positive findings, there is evidence that fails to support enhanced EF in bilinguals (e.g., Hilchey & Klein, 2011). A study by Paap and Greenberg (2013) tested the bilingual advantage in inhibitory control, monitoring, and switching in young adults. Their results showed little support for an executive processing advantage related to bilingualism and implied that in previous studies, the use of one task to evaluate EF might have led to a misinterpretation of findings. The authors also stressed the importance of adequately matching the study groups. In another study, Paap, Anders-Jefferson, Mikulinsky, Masuda, and Mason (2019) did not find a bilingual advantage in four tasks of inhibitory control, and the authors highlighted the possibility that bilingualism is task-specific and part of a language-processing system. Furthermore, Antón, Carreiras, and Duñabeitia (2019) did not find a bilingual EF advantage in young adults using a large sample of monolingual and bilingual participants who underwent extensive EF testing.

A study by Sörman, Hansson, and Ljungberg (2019) included two distinct adult bilingual samples to explore the effects of bilingualism and linguistic distance (Swedish–Finnish and Swedish–English bilinguals) on cognitive function. Results did not indicate a bilingual effect, and linguistic distance similarly failed to impact cognitive control. In a meta-analysis, Lehtonen, Soveri, Laine, Järvenpää, de Bruin, and Antfolk (2018) considered a wide range of moderating variables (e.g., task paradigm, testing language, and group matching) and concluded that publication bias might be responsible for the associations between bilingualism and EF advantages.

Bilingualism has also been associated with disadvantages on verbal tests (e.g., fluency tests; Gollan, Montoya & Werner, 2002; Lehtonen et al., 2018), presumably as a result of the increased linguistic interference between languages (Rosselli, Ardila, Araujo, Weekes, Caracciolo, Padilla & Ostrosky-Solís, 2000) and reduced exposure to each language when both are used comparably (Lehtonen et al., 2018).

Considering the effects of bilingualism as “advantages” or “disadvantages” has been deemed inaccurate and reductionistic (Leivada, Westergaard, Duñabeitia & Rothman, 2020). A shift to focus on the mechanisms and the variables underlying any bilingual adaptations/ameliorations (e.g., cognitive vs. brain reserve) has been proposed.

### Bilingualism and reserve

Bilingualism may contribute to cognitive (Stern, 2009) or brain reserve (Katzman, 1993). Cognitive reserve is an active and modifiable mechanism by which the brain attempts to cope in cases of changes or damage through the use of preexisting cognitive processes (Stern, 2009; Cognitive abilities, education, occupation, exercise, or social engagement are experiences that may contribute to the development of cognitive reserve (Stern, Arenaza-Urquijo, Bartrés-Faz, Belleville, Cantilon, Chetelat, Ewers, Franzmeier, Kempermann, Kremen, Okonkwo, Scarmeas, Soldan, Udeh-Momoh, Valenzuela, Vemuri, Vuoksimaa & the Reserve, Resilience and Protective Factors PIA Empirical Definitions and Conceptual Frameworks Workgroup, 2020). Brain reserve is a passive mechanism attempting to cope with damage and is derived from neuronal count or overall brain volume; the functional capacity to deal with brain injury (e.g., neurodegenerative diseases) varies across individuals (Stern, 2009). An additional distinction between the types of reserve relates to the aim of the activity. For instance, active attempts to increase reserve, such as exercise differs from passive attempts (e.g., education) wherein the individual participates in the activity without the goal of increasing reserve. Accordingly, bilingualism may be considered passive; individuals may have learned a second language because of their circumstances. However, the use of two languages in daily life eventually necessitates active control (Bialystok, 2021). Greater demands on language control systems in bilinguals might increase reserve, leading to a delay in the onset of neurodegenerative clinical conditions such as dementia (Bialystok, Craik, Binns, Ossher & Freedman, 2014a).

Cognitive reserve in bilinguals may result from an interaction between brain and behavioral adaptations resulting from the active and constant use of two languages (Grant, Dennis & Li, 2014). The brain bases would correspond to quantifiable neuroanatomical modifications in gray matter volume (GMV), white matter volume (WMV), or cortical thickness. The behavioral bases would manifest as performance changes in tasks that depend on EF, attention, and switching. The extrapolation effect from language (specific) to other cognitive EF (domain-general) suggests an overlap of the cognitive control and language control networks (Abutalebi & Green, 2016). The mechanisms underlying the overlap of functions is unknown; however, it may result from bilinguals’ increased switching practice. Changes in bilinguals’ complexity of EEG neural networks (greater sample entropy) with greater brain signal complexity are believed to index the ability to rapidly switch between brain states (Grundy, Anderson & Bialystok, 2017). The cortical vs. subcortical and gray matter vs. white matter structural brain adaptations of bilingualism may be associated with the type of dual language experience, and individual factors within bilingual groups should be considered. For instance, initial exposure to a second language could cause gray matter cortical and subcortical changes, whereas increased experience in highly immersed bilinguals is related to additional structural changes in brain connectivity and subcortical regions (Pliatsikas, 2020). Furthermore, researchers have also suggested that varying ages of second language acquisition may result in different adaptations when learning new tasks, and overall, the importance of considering individual differences and examining bilingualism across development is emphasized (Hernandez, Claussenius-Kalman, Ronderos & Vaughn, 2018; Hernandez, Claussenius-Kalman, Ronderos, Castilla-Earls, Sun, Weiss & Young, 2019).

As a result of higher cognitive reserve, bilinguals may display symptoms of dementia at a later age than monolinguals (Fischer & Schweizer, 2014; Perani & Abutalebi, 2015; Perani et al., 2017). For example, Alladi, Bak, Duggirala, Surampudi, Shailaja, Shukla, and Kaul (2013) reported that in their sample of bilinguals (some spoke more than two languages), there was a 4.5-year delay compared to monolinguals in the onset of AD, frontotemporal, and vascular dementia, after controlling for education. The bilingual group in this study reported speaking a wide range of languages, and as emphasized by Paap, Johnson, and Sawi (2016), it included a higher proportion of men and individuals from an urban setting with higher education. Similarly, Bialystok, Craik, and Freedman (2007) reported a four-year delay in dementia symptoms for bilinguals compared to monolinguals. Lastly, Woumans, Santens, Sieben, Versijpt, Stevens, and Duyck (2015) reported that bilinguals exhibited a delay of 4.6 years in symptom manifestation and 4.8 years in AD diagnosis compared to monolinguals.

The mechanism underlying the delay in dementia diagnosis for bilinguals may result due to a compensatory strengthening of brain circuits involved in executive control (EC), which in turn strengthens frontostriatal and frontoparietal networks (Gold, 2015). This explanation, therefore, lends support to the EF and cognitive reserve bilingual advantages, with a strong EF-bilingualism association acting as the underlying mechanism in the bilingualism-reserve link. The additional effort put forth by bilinguals to control their languages may be part of a general EC system, explaining the enhanced EF in bilinguals. Consequently, these enhanced abilities would compensate for the missing resources resulting from a neurodegenerative disease.

Consistent with the theory of cognitive reserve, aging bilinguals exhibit increased damage in several brain regions. Schweizer, Ware, Fischer, Craik, and Bialystok (2012) found that bilinguals with AD had higher brain atrophy in the temporal horn (an area used to distinguish AD patients from healthy adults) compared to a group of matched monolinguals. Recently, Costumero, Marin-Marin, Calabria, Belloch, Escudero, Baquero, Hernandez, Ruiz de Miras, Costa, Parcet, and Ávila (2020) compared matched samples of monolinguals (Spanish) and bilinguals (Spanish–Catalan) and found that MCI bilinguals exhibited higher brain atrophy despite similar performance on cognitive tests. These differences were found in the lingual and supramarginal gyri, which are typically affected in AD (Schwindt & Black, 2009).

Recently, Heim, Stumme, Bittner, Jockwitz, Amunts, and Caspers (2019) examined GMV in the inferior parietal lobe (IPL) and inferior frontal gyrus (IFG) in a large sample of monolingual (*n* = 224) and bilingual (*n* = 175) participants. Results suggested that bilinguals had higher GMV in the left IPL and left IFG than monolinguals, but only at a younger age, with the normal decline associated with aging occurring faster in bilinguals but differing across regions, appearing later in the IPL than the IFG. The authors concluded that the increased ‘reserve’ in linguistic areas diminished with age at a faster pace than the ‘reserve’ in nonlinguistic areas. Duncan, Nikelski, Pilon, Steffener, Chertkow, and Phillips (2018) reported similar findings when comparing MCI and AD multilingual (of which over half were bilinguals, and the rest spoke three or more languages) and monolingual participants. Multilingual AD patients had reduced cortical thickness and lower tissue density in AD-related regions (implying higher cognitive reserve), as well as higher GMV in areas associated with language and cognitive control (e.g., bilateral IFG and right ventromedial prefrontal cortex, among others). Of note, this study obtained similar results with a non-immigrant MCI sample; however, these researchers did not include a healthy control group. Finally, Costumero et al. (2020) also reported lower GMV in MCI bilinguals compared to monolinguals in the lingual and supramarginal gyri (brain regions known to be affected by AD).

Despite these findings, several studies fail to support an association between bilingualism and cognitive reserve. Crane, Gibbons, Arani, Nguyen, Rhoads, McCurry, Launer, Masaki, and White (2009) found that second language (L2) writing fluency did not protect against cognitive decline. Similarly, Yeung, St John, Menec, and Tyas (2014) did not find a link between bilingualism and dementia risk in a 5-year longitudinal study. Zahodne et al. (2014) could not determine a protective effect of bilingualism on cognitive decline or in the conversion to dementia in bilingual immigrants. Nevertheless, bilinguals performed better at baseline on memory and EF tasks. Finally, Mungas et al. (2018) did not report a relationship between bilingualism and the rate of cognitive decline in a large longitudinal study with a sample of monolinguals and bilinguals.

### Study aims

In general, research appears to suggest that the beneficial effects of bilingualism in aging are associated with its protective and enhancing influence over brain networks and regions related to EF and language (Luk, Bialystok, Craik & Grady, 2011). Numerous studies do not support a delay in the onset of symptoms or dementia diagnosis, and instead, emphasize the inconsistent findings and methodological concerns (Mukadam, Sommerlad & Livingston, 2017; Yeung et al., 2014).

The present study sought to include aspects of bilingualism research that have been emphasized by investigators, and to overcome previous limitations as follows: a) the inclusion of the age of acquisition of the L2 (Luk et al., 2011); b) ensuring that all bilingual participants were proficient in the same languages; c) the inclusion of a monolingual control group; d) utilizing longitudinal data to explore changes associated with disease progression (Calvo, García, Manoiloff & Ibáñez, 2016); e) the use of more than one EF measure derived from separate tasks to minimize the possibility that performance differences are task-specific (Paap, Johnson & Sawi, 2015); and f) the inclusion of neuroimaging assessments (van den Noort, Vermeire, Bosch, Staudte, Krajenbrink, Jaswetz, Struys, Yeo, Barisch, Perriard, Lee & Lim, 2019), particularly of structural neuroimaging that could identify brain differences between bilinguals and monolinguals while removing the influence of task-related factors (García-Pentón, Fernandez Garcia, Costello, Duñabeitia & Carreiras, 2016).

This research analyzed differences between monolinguals and bilinguals classified as cognitively normal (CN) or diagnosed with Mild Cognitive Impairment (MCI) or dementia in the GMV of memory-related regions and frontal regions associated with EF and language. Additionally, the effects of bilingualism on EF scores during two visits in cognitively normal (CN) and Mild Cognitive Impairment (MCI) participants were examined. Four EF tasks were used: Digit Span Backwards (Wechsler, 2014a); Trail Making Test difference score (Reitan & Wolfson, 1986, 1993); Stroop Color-Word Interference (Stroop, 1935; Trenerry, Crosson, DeBoe & Leber, 1989), and category fluency average scores. To move beyond monolingual and bilingual comparisons, a Bilingualism Index (BI) and the age of acquisition of English (within the Spanish–English bilingual sample) were used to predict EF performance and GMV.

Consistent with previous findings (Duncan et al., 2018), it was predicted that bilinguals would exhibit greater GMV in bilateral EF and language regions, but a higher degree of GMV loss in memory-related regions (for the MCI and dementia groups). Additionally, it was expected that bilinguals would outperform monolinguals on EF tasks except those with a strong verbal component (e.g., category fluency), as research suggests that the crosslanguage interference is associated with lower scores on verbal tasks (Rosselli et al., 2000). Additionally, a BI value representing a balance in linguistic proficiency was expected to be the most significant predictor of EF performance, as previous findings support an association between these individual bilingual components and EF performance (Rosselli, Loewenstein, Curiel, Penate, Torres, Lang, Greig, Barker & Duara, 2019; Yow & Li, 2015). Lastly, bilinguals were expected to be older at the onset of cognitive symptoms, as was suggested by previous reports (Alladi et al., 2013; Woumans et al., 2015).

## Method

### Participants

Recruitment took place during memory screenings and events at community senior centers. Participants were part of the 1Florida Alzheimer’s Disease Research Center (ADRC), a 5-year longitudinal IRB-approved study (Mount Sinai Medical Center-IRB) that began in 2015 at Mount Sinai Medical Center in Miami Beach, Florida. Two samples were included: a) CN, MCI, or dementia participants with Magnetic Resonance Imaging (MRI) data; and b) CN or MCI participants with longitudinal neuropsychological data. Table 1 provides the demographic characteristics of the imaging and longitudinal samples. Participants were included if they were born in the US or a Spanish-speaking Latin American country.

The first sample included 214 participants with MRI data (*M*_*age*_ = 71.21, *SD* = 7.23), of which 75 were CN, 106 were diagnosed with MCI, and 33 with dementia (see Diagnostic Criteria below). Within this sample, 124 participants were classified as bilinguals; 115 of these reported Spanish as their first language (L1) and English as L2 (Spanish–English bilinguals), 8 were English–Spanish bilinguals, and one was a simultaneous bilingual. Out of the 90 monolingual participants, 72 were English monolinguals, and 18 were Spanish monolinguals (see below for a description of the language groups). The diagnostic groups did not differ in the monolingual and bilingual distribution, *χ*^2^ (2, *N* = 214) = .59, *p* = .75.

The 72 English monolinguals and 13 bilinguals were born in the US (39.72% of the sample). The rest of the sample comprised 111 bilinguals who were immigrants from the following countries (including Puerto Rico): Cuba (37.85%), Colombia (9.81%), Argentina (3.74%), Nicaragua (1.87%), Venezuela (1.40%), Puerto Rico (1.40%), Peru (1.40%), Chile (0.93%), Ecuador (0.93%), Dominican Republic (0.47%), and Guatemala (0.47%). The average age of immigration to the US was 25.51 (*SD* = 16.47), and these individuals had lived in the US for an average of 45.55 years (*SD* = 15.83). For the Spanish monolingual group, the average age of immigration to the US was 36.28 (*SD* = 11.25), with an average duration of residence of 35.44 years (*SD* = 11.97). The bilingual group reported an average age of immigration of 23.76 (*SD* = 16.55) years, and this group had lived in the US for an average of 47.19 years (*SD* = 15.82).

Participants’ occupations were compared across the language groups to examine potential socioeconomic differences. Occupations were classified according to Szreter’s (1984) scale as follows: Unskilled Manual Labor, Skilled Manual Labor, or Professional Labor. These data were available for 149 participants; 85.9% of participants reported occupations of Professional Labor, 10.1% were determined to have performed Skilled Manual Labor, and 4% were classified as having performed Unskilled Labor. Occupational categories did not differ between the monolingual and bilingual groups, *p* > .05.

For the longitudinal analyses, 171 participants (64.9% female) with Visit 1 (V1) and Visit 2 (V2) neuropsychological evaluations were initially included. This subsample comprised 66 CN, 87 MCI, and 18 dementia participants. Within the dementia group, there were six monolingual and 12 bilingual participants. Due to the limited number of available V2 data for the dementia sample and the uneven language group distribution, this diagnostic group was excluded from the longitudinal analyses. The final sample included 153 participants (64.7% female; 66 CN and 87 MCI) with a mean age of 70.97 (*SD* = 6.93) and 63 monolinguals and 90 bilinguals. Within the bilingual group, there were 83 Spanish–English bilinguals and 7 English–Spanish bilinguals. Within the monolingual group, 50 were monolingual English speakers, and 13 were Spanish monolinguals.

Participants were assessed with a comprehensive neuropsychological battery, and for eligible participants, MRI scans were completed at Mount Sinai Medical Center during V1. Participants were seen by a clinician and were accompanied by an informant. With the informant’s report, the clinician answered the following question: “Based on the clinician’s judgment, at what age did the cognitive decline begin?”. All participants and their informants gave informed consent.

The time between V1 and V2 ranged from 10 to 33 months (*M* = 14.04, *SD* = 3.34). This variable did not differ between diagnostic or language groups, *p* > .05. Moreover, Spearman’s correlations suggested that the visit interval was not associated with neuropsychological change scores from V2 and V1; therefore, it was not included in the analyses.

#### Exclusion criteria

Participants who met the following criteria were excluded: a) presence of major psychiatric disorders such as psychosis, bipolar, or unipolar disorders; b) missing Language Experience Acquisition Proficiency Questionnaire (LEAP-Q; Marian, Blumenfeld & Kaushanskaya, 2007) data; and c) first and second languages besides English or Spanish.

#### Diagnostic criteria

Groups were carefully classified by the following diagnostic criteria as outlined in our previous papers (Loewenstein, Curiel, DeKosky, Rosselli, Bauer, Grieg-Custo, Penate, Li, Lizagarra, Golde, Adjouadi & Duara, 2017; Rosselli et al., 2019; Torres, Rosselli, Loewenstein, Curiel, Vélez Uribe, Lang, Arruda, Penate, Vaillancourt, Greig, Barker, Bauer & Duara, 2019) and which has been adopted as stringent and standard diagnostic criteria (Brooks & Loewenstein, 2010).

#### Cognitively Normal (CN)

The CN group and their collateral informants did not report memory deficits, cognitive decline, or any impairment in daily function after an extensive interview, including the Global Clinical Dementia Rating Scale (CDR; Morris, 1993). All CN participants received a Global CDR score of 0. Also, the CN group received additional standard neuropsychological assessment and had standard neuropsychological measure scores less than 1 SD below expected levels related to age, education, and language-related norms on the following measures: a) Delayed Recall of the Hopkins Verbal Learning Test-Revised (HVLT-R; Brandt, 1991; Cherner, Suarez, PLazzaretto, Fortuny, Mindt, Dawes, Marcotte, Grant & Heaton, 2007), b) category and letter fluency (Benton & Hamsher, 1976), c) Block Design of the Wechsler Adult Intelligence Scale (WAIS-IV; Wechsler, 2014a, 2014b), and d) Trail-Making Test B (TMT-B; Reitan & Wolfson, 1993).

#### Mild Cognitive Impairment

The MCI group met Petersen’s criteria (Petersen, 2004), had memory complaints confirmed by a reliable collateral informant after an extensive interview with an experienced examiner including the CDR.

To meet the criteria for MCI, the participant had to obtain a Global CDR score of .5 and meet the criteria for a Minor Neurocognitive disorder by Diagnostics and Statistical Manual of Mental Disorders (DSM-5; American Psychiatric Association, 2013), including the lack of social and/or functional impairment. In addition, MCI patients had independent neuropsychological testing and had scores on the HVLT-R (Brandt, 1991) or NACC story delayed recall (Weintraub, Salmon, Mercaldo, Ferris, Graff-Radford, Chui, Cummings, DeCarli, Foster, Galasko, Peskind, Dietrich, Beekly, Kukull & Morris, 2009) of 1.5 SD or greater, below what is expected using the same normative data listed for the CN group above. Other non-memory measures (e.g., TMT-B and category fluency) could be 1.5 SD or greater, above or below the mean.

#### Dementia

Individuals diagnosed with dementia had the same extensive clinical interview described above with a CDR Global score of 1.0, met criteria for a Major Neurocognitive disorder by Diagnostics and Statistical Manual and Mental Disorders (DSM-5; American Psychiatric Association, 2013), and demonstrated impairment in social or occupational function. In addition, scores were more than 2 SD below the mean relative to age, education, and language-related norms, as described above. The dementia group also met the criteria for Major Neurocognitive disorder, and clinically, this group also met the criteria for probable AD (McKhann, Knopman, Chertkow, Hyman, Jack, Kawas, Klunk, Koroshetz, Manly, Mayeux, Mohs, Morris, Rossor, Scheltens, Carrillo, Thies, Weintraub & Phelps, 2011).

### Materials

#### Language Experience and Proficiency Questionnaire (LEAP-Q)

The LEAP-Q (Marian et al., 2007) was used to create monolingual and bilingual groups for language group comparisons. If a participant reported an average English or Spanish proficiency of 3 (“fair”) or more in speaking, understanding, and reading, they were considered bilingual; otherwise, they were classified as monolinguals. The order and age of language acquisition were determined from participant responses.

A Bilingualism Index (BI) and the age of acquisition of L2 of Spanish–English bilingual participants were used as variables related to bilingualism.

#### Bilingualism Index (BI)

The lower average LEAP-Q proficiency score (speaking, understanding, and reading in one language, English, or Spanish) was divided by the higher average LEAP-Q proficiency score (speaking, understanding, and reading in the other language). Participants rated their proficiency on a 0 to 10 Likert scale (0 = none, 1 = very low, 2 = low, 3 = fair, 4 = slightly less than adequate, 5 = adequate, 6 = slightly more than adequate, 7 = good, 8 = very good, 9 = excellent, 10 = perfect). The index resulted in scores ranging from zero (monolingual) to one (bilingual), providing information about the balance of an individual’s bilingual abilities.

Rosselli et al. (2019) used the same measure in their study, and Gollan, Salmon, Montoya, and Galasko (2011) originally developed a similar BI using scores from the Boston Naming Test (Kaplan, Goodglass & Weintraub, 1983), dividing the proportion of pictures named correctly in one language by the proportion of pictures named correctly in the other language.

#### Digit Span Backwards

Participants are read a sequence of one-digit numbers and asked to repeat the sequence in the reverse order. The Digit Span Backwards is considered a task of EF, specifically, working memory (WM; Hilbert, Nakagawa, Puci, Zech & Bühner, 2015; Miyake, Friedman, Emerson, Witzki, Howerter & Wager, 2000).

#### Trail Making Test (TMT)

The TMT consists of parts A and B. During Part A, participants are instructed to connect 25 circles numbered from 1 to 25 as quickly as possible. During Part B, participants are asked to connect circles with numbers and letters while alternating and maintaining numerical and alphabetic order (Reitan & Wolfson, 1986). Participants are allowed 150 s for part A and 300 s for part B. Errors are corrected by the experimenter as soon as they occur. The time to complete TMT-B minus the time to complete TMT-A (TMT-B minus TMT-A) was used, as this difference score has been suggested to assess cognitive flexibility and switching without considering dexterity (Corrigan & Hinkeldey, 1987; Kopp, 2011; Reitan, 1958).

#### Stroop Color-Word Interference Test

A measure of inhibitory control and interference, the Stroop test (Stroop, 1935; Trenerry et al., 1989), requires participants to inhibit reading a word (a color) while correctly identifying the ink color of the text. Participants completed color (C), word (W), and color-word conditions (CW), with 45 s given for each. Predicted CW scores were calculated with the following formula: (WxC)/(W+C) and subtracted from the CW score. Stroop interference scores indicate the degree to which the participant can control interference.

#### Category Fluency

Participants are instructed to name as many animals, fruits, and vegetables as possible during 60 s per category. Incorrect words include proper names, numbers, repetitions, or words sharing similar roots. The total score of the three categories was averaged. This task involves language and EF, and lower scores are reported in AD than normal controls (Weakley & Schmitter-Edgecombe, 2014).

### Procedure

Monolingual European American participants were evaluated in English, and monolingual Hispanic participants were tested in Spanish. Bilingual participants selected their preferred language of evaluation (English or Spanish). In these cases, the neuropsychological examination was completed by fluent English–Spanish bilingual psychometricians. Out of 135 Hispanic participants, 71.9% were evaluated in Spanish. Please see Lang, Rosselli, Greig, Torres, Vélez-Uribe, Arruda, Barker, Garcia, Loewenstein, Curiel, and Duara (2021) for a complete description of the neuropsychological battery.

Spanish evaluations were completed with equivalent standardized neuropsychological tests and had appropriate age, education, and language normative data for the translated versions (Arango-Lasprilla, Rivera, Aguayo, Rodríguez, Garza, Saracho, Rodríguez-Agudelo, Aliaga, Weiler, Luna, Longoni, Ocampo-Barba, Galarza-Del-Angel, Panyavin, Guerra, Esenarro, García de la Cadena, Martínez & Perrin, 2015a; Arango-Lasprilla, Rivera, Garza, Saracho, Rodríguez, Rodríguez-Agudelo, Aguayo, Schebela, Luna, Longoni, Martínez, Doyle, Ocampo-Barba, Galarza-Del-Angel, Aliaga, Bringas, Esenarro, García-Egan & Perrin, 2015b; Benson, de Felipe, Xiaodong & Sano, 2014; Golden, 1999; Gollan, Weissberger, Runnqvist, Montoya & Cera, 2012; Ostrosky-Solís, López-Arango & Ardila, 2000; Peña-Casanova, Quiñones-Ubeda, Gramunt-Fombuena, Aguilar, Casas, Molinuevo, Robles, Rodríguez, Barquero, Antúnez, Martínez-Parra, Frank-García, Fernández, Molano, Alfonso, Sol, Blesa & NEURONORMA Study Team, 2009a; Peña-Casanova, Quiñones-Ubeda, Gramunt-Fombuena, Quintana, Aguilar, Molinuevo, Serradell, Robles, Barquero, Payno, Antúnez, Martínez-Parra, Frank-García, Fernández, Alfonso, Sol, Blesa & NEURONORMA Study Team, 2009b; Peña-Casanova, Quiñones-Úbeda, Gramunt-Fombuena, Quintana-Aparicio, Aguilar, Badenes, Cerulla, Molinuevo, Ruiz, Robles, Barquero, Antúnez, Martínez-Parra, Frank-García, Fernández, Alfonso, Sol & Blesa, 2009c; Wechsler, 2014b).

#### Imaging

MRI images were obtained using a Siemens Medical System Skyra 3 Tesla Scanner with Software version: “syngo MR E11”. Coil: “Siemens Head/Neck 20”. The Scanning Sequences used were 3D T1-W Magnetization-Prepared Rapid Gradient-Echo (MPRAGE.) Sagittal, three-dimensional images with resolution of 1 mm (~12 min., TR = 2150 ms, TE = 4.38 ms, TI = 1100 ms, 160 slices, 1 × 1 × 1 mm^3^) obtained from approximately 1 cm left of the skull to 1 cm right of the skull, allowing room for spatial reorientation along with defined anatomic coordinates. We employed visual inspection of the segmentation as outlined in the Alzheimer’s Disease Neuroimaging Initiative protocol. No segmentation issues were noted, and manual adjustment was not required. Brain parcellation was obtained utilizing a 3D T1-weighted sequence (Magnetization-Prepared Rapid Gradient-Echo -MPRAGE) with 1.0 mm isotropic resolution. FreeSurfer Version 5.3 software (Desikan, Segonne, Fischl, Quinn, Dickerson, Blacker, Buckner, Dale, Maguire, Hyman, Albert & Killiany, 2006) was used (Loewenstein et al., 2017).

No preprocessing is done on the MRI images, except for quality control carried out to check for shading which was corrected in some MRIs using an augmented statistical parametric method (SPM). The few MRIs that were affected by biased field were discarded from further analysis. Our datasets consisting of 3D MRIs through our web interface pipeline proved successful with regards to the Global Alzheimer’s Association Interactive Network (GAAIN). To further validate our Neuro-Imaging Web Services Interface (NWSI) pipeline, the GAAIN centiloid initiative’s data has been passed through the pipeline to ensure that the results are comparable to further improve the reach and usability of the centiloid data produced by NWIS. This entails comparing each portion of the process such as the DCM to NIfTI (NII) conversion, the MRI segmentation (GAAIN opted for SPM instead of FreeSurfer), the MRI registration.

Whole-brain grey matter combines multiple brain regions and is not useful for evaluating relationships with neuropsychological tests. In contrast, including all brain regions obtained from FreeSurfer as dependent measures would lead to an excess of comparisons. Given the specific neuropsychological measures employed and the evaluation of individuals at high risk of developing AD, we examined left and right brain regions from MRI scans that are associated with memory (hippocampi and entorhinal cortex; (Henneman, Sluimer, Barnes, van der Flier, Sluimer, Fox, Scheltens, Vrenken & Barkhof, 2009; Leandrou, Mamais, Petroudi, Kyriacou, Reyes-Aldasoro & Pattichis, 2018), EF (orbitofrontal cortex [OFC]; Bryden & Roesch, 2015), and language (inferior frontal gyrus [IFG]; Duncan et al., 2018). For the OFC, the medial and lateral OFC were included. For the IFG, the pars opercularis, pars orbitalis, and pars triangularis were included. All GMV measurements were corrected for total individual intracranial volume.

### Statistical analyses

IBM SPSS 25 was used. Two one-way Welch’s ANOVAs were used to compare English and Spanish proficiency between the language groups, as the Levene statistic indicated unequal variances. Within the bilingual group, a Repeated Measures General Linear Model (GLM) was used to compare English and Spanish proficiency.

Six 2 (Language Group) x 3 (Diagnostic Group) univariate GLM analyses were used to compare the language and diagnostic groups on the GMV of bilateral regions associated with language (left and right IFG), EF (left and right OFC), and memory (left and right hippocampi and entorhinal cortices). Age, education, and sex were included as covariates. The Bonferroni correction was used to adjust for multiple comparisons; this resulted in alpha values of .025 for the two language regions and the two EF regions, and .013 for the four memory regions.

Four 2 × 2 × 2 mixed factorial GLM (2 between and 1 within factors) were used to compare the performance of the language (monolingual vs. bilingual) and diagnostic groups (CN and MCI) on the following neuropsychological tests over two visits: Digit Span Backwards (Wechsler, 2014a), TMT-B minus TMT-A (Reitan & Wolfson, 1993), Stroop interference (Stroop, 1935; Trenerry et al., 1989), and category fluency average scores. Age, education, and sex were included as covariates.

Spearman’s rank correlations were used to explore the relationship between the EF scores during V1 and the GMV of left and right regions related to memory, language, and EF. Due to unequal language group sizes, Fisher r-to-z transformations were conducted to test whether the correlation coefficients differed between the groups (VassarStats; Lowry, n.d.).

The predictive value of two variables related to bilingualism (Bilingualism Index [BI] and the English age of acquisition) was assessed using linear regressions in Spanish–English bilinguals. These analyses were conducted for EF tests and in GMV areas that exhibited language group differences in the previous GLM analyses. The bilingualism variables were used as predictors of the following: a) EF test performance in three tasks (Digit Span Backwards, Stroop interference, and category fluency; b) GMV in left IFG; and c) GMV in left and right OFC. These analyses included age, education, sex, language, and diagnostic group as predictors.

One 2X3 Univariate GLM compared the language groups using the age at which cognitive symptoms began. Age, education, and sex were included as covariates. Additionally, four linear regression analyses were used to examine the predictive value of GMV (IFG, OFC, hippocampi, and entorhinal cortices) and language group over the age of cognitive decline. A linear regression model was used to examine the relationship between GMV in areas that exhibited language group differences and Stroop interference scores (Del Maschio, Sulpizio, Gallo, Fedeli, Weekes & Abutalebi, 2018). Language group, age, education, sex, and normalized left IFG GMV were included as predictors of Stroop interference scores.

Fisher’s Least Significant Difference (LSD) post hoc analyses were used.

## Results

### Language proficiency (LEAP-Q) of monolinguals and bilinguals

The average English and Spanish proficiency levels of the monolingual and bilingual groups did not differ. Within the bilingual group, Spanish proficiency was significantly higher (*M* = 9.16, *SD* = 1.21) than English proficiency (*M* = 8.32, *SD* = 1.66), *F*(1,123) = 19.25, *p* < .001, $\eta_{p}^{2}=.135$, with an average BI of .83 (*SD* = .16).

### Language group

#### Imaging

For the imaging analyses, the diagnostic groups differed in sex, *χ*^2^ (2, *N* = 214) = 9.22, *p* = .010, with a greater number of females in all groups. There were also group differences in education, *F*(2,211) = 3.87, *p* = .022, with the CN group reporting the highest years of education, followed by the MCI group, and lastly, the dementia group. No diagnostic group differences were found in age, ethnicity, or language group. The language groups differed in age, *F*(1,212) = 4.59, *p* = .033, with the monolingual group (*M*_age_ = 72.49, *SD* = 7.91) older than the bilingual group (*M*_age_ = 70.27, *SD* = 7.12). Education, sex, or diagnosis did not differ across groups.

The language groups were compared on the GMV of regions related to memory (hippocampi and entorhinal cortices), language (inferior frontal gyri; IFG), and EF (orbitofrontal cortices; OFC). See Table 2.

Two 2X3 Univariate GLM with demographic variables as covariates were used to compare bilinguals and monolinguals on the GMV of left and right IFG across the three diagnostic groups. The only significant main effect was found between the language groups, *F*(1,205) = 11.88, *p* = .001, $\eta_{p}^{2}=.055$, with bilinguals exhibiting greater left IFG GMV than monolinguals. The main effect of diagnosis and the interaction between the language and the diagnostic groups were not significant, and age was a significant covariate, *p* < .001, whereas education and sex were not.

There was a main effect of language group in the GMV of the right IFG, *F*(1, 205) = 4.42, *p* = .037, $\eta_{p}^{2}=.021$, with bilinguals exhibiting larger volumes than monolinguals; however, this comparison lost significance after the Bonferroni correction. There was a significant main effect of diagnostic group, *F*(2,205) = 3.94, *p* = .021, $\eta_{p}^{2}=.037$. Post hoc analyses using Fisher’s LSD indicated that the dementia group had lower IFG volume in the right hemisphere compared with the other two groups, *ps* < 0.05. No significant differences were observed between the MCI and CN groups. The interaction between diagnostic and language groups was not significant. Age and sex were significant covariates, *p* < .001.

For the OFC, two 2 × 3 Univariate GLM were used to compare the GMV of these regions between the language and diagnostic groups. A main effect of language group was found in the right and left OFC, *F*(1,205) = 11.52, *p* = .001, $\eta_{p}^{2}=.053$, and *F*(1, 205) = 8.56, *p* = .004, $\eta_{p}^{2}=.04$, respectively. Bilinguals exhibited higher GMV in these regions compared to monolinguals. In the right and left OFC, a main effect of diagnostic group was also found, *F*(2,205) = 9.92, *p* < .001, $\eta_{p}^{2}=.09$, and *F*(2,205) = 9.59, *p* < .001, $\eta_{p}^{2}=.09$, respectively. Post hoc analyses using Fisher’s Least Significant Difference (LSD) indicated that the dementia group had the lowest GMV in the OFC of the right and left hemispheres compared to the other two groups, *ps* < .005. Significant interactions between the language and diagnostic groups were found in the right and left OFC, *F*(2,205) = 7.14, *p* = .001, $\eta_{p}^{2}=.07$, and *F*(2,205) = 5.73, *p* = .004, $\eta_{p}^{2}=.05$, respectively. Post hoc analyses indicated that the finding of higher GMV in the bilingual group compared with the monolingual group in the left and right OFC was significant only within the dementia group, *ps* < .05. Age was the only significant covariant of the right and left OFC, *p* = .001, and *p* < .001, respectively.

In addition to the language and EF areas, two 3 × 2 multivariate GLM were used to compare the language and diagnostic groups in the GMV of bilateral hippocampi and entorhinal cortices. No significant language group effects were found on either hemisphere for either region. In the left and in right hippocampi, there was a main effect of diagnostic group, *F*(2,205) = 22.73, *p* < .001, $\eta_{p}^{2}=.18$, and *F*(2, 205) = 18.71, *p* < .001, $\eta_{p}^{2}=.15$, respectively. Post hoc analyses indicated the dementia groups had the lowest GMV of bilateral hippocampi compared with the other groups, *ps* < .005, and the CN and MCI groups also differed significantly from each other, *p* < .001. Age and sex were significant covariates, *ps* < .001.

There was a main effect of diagnostic group in the GMV of the left and right entorhinal cortices, as well, *F*(2, 205) = 12.44, *p* < .001, $\eta_{p}^{2}=.11$, and *F*(2, 205) = 9.91, *p* < .001, $\eta_{p}^{2}=.09$, respectively. LSD post hoc analyses indicated that the left and right entorhinal cortex were significantly larger in the CN compared to the MCI and the dementia groups, *ps* < .001. The GMV of the left entorhinal cortex also differed significantly between the CN and MCI groups, *p* = .038. Age was a significant covariate, *p* < .001. No significant interactions between the language and diagnostic groups were found in these regions.

#### Associations between gray matter volume and executive function

For validity purposes, Spearman correlations were used to examine the relationship between the volume of the regions of interest (ROIs) and neuropsychological performance across the language groups. See Tables 3–6.

For the monolingual group, significant positive correlations emerged between the Digit Span Backwards and the left IFG and between category fluency and bilateral hippocampi, entorhinal cortices, IFG, and OFC. In the bilingual group, the TMT-B minus TMT-A was negatively correlated with the GMV of the bilateral hippocampi, entorhinal, and OFC, and there were positive correlations between fluency and bilateral hippocampi, entorhinal, and left OFC. Stroop interference scores were not correlated with these regions.

Due to different sample sizes between the language groups, Fisher r-to-z transformations were performed. These analyses suggested that the TMT difference score correlation coefficients were not significantly different between monolinguals and bilinguals for the left hippocampus, right entorhinal cortex, or bilateral OFC. Differences were significant between the correlation coefficients from the right hippocampus, *p* = .03, and the left entorhinal cortex, *p* = .04.

### Longitudinal analyses

For the longitudinal neuropsychological analyses, 153 participants (90 bilinguals) diagnosed as CN or MCI were included. The diagnostic groups were similar in age and education, but differences were found in sex, *χ*^2^ (1, *N* = 153) = 4.62, *p* = .032. The language groups did not differ in age, education, or sex. See Table 7 for the neuropsychological scores across diagnostic and language groups.

Four 2 × 2 × 2 mixed factorial GLM were used to compare the performance of the language and diagnostic groups over two time points on the Digit Span Backwards, Stroop interference, category fluency average, and TMT-B minus TMT-A scores.

**Fig. 1.**
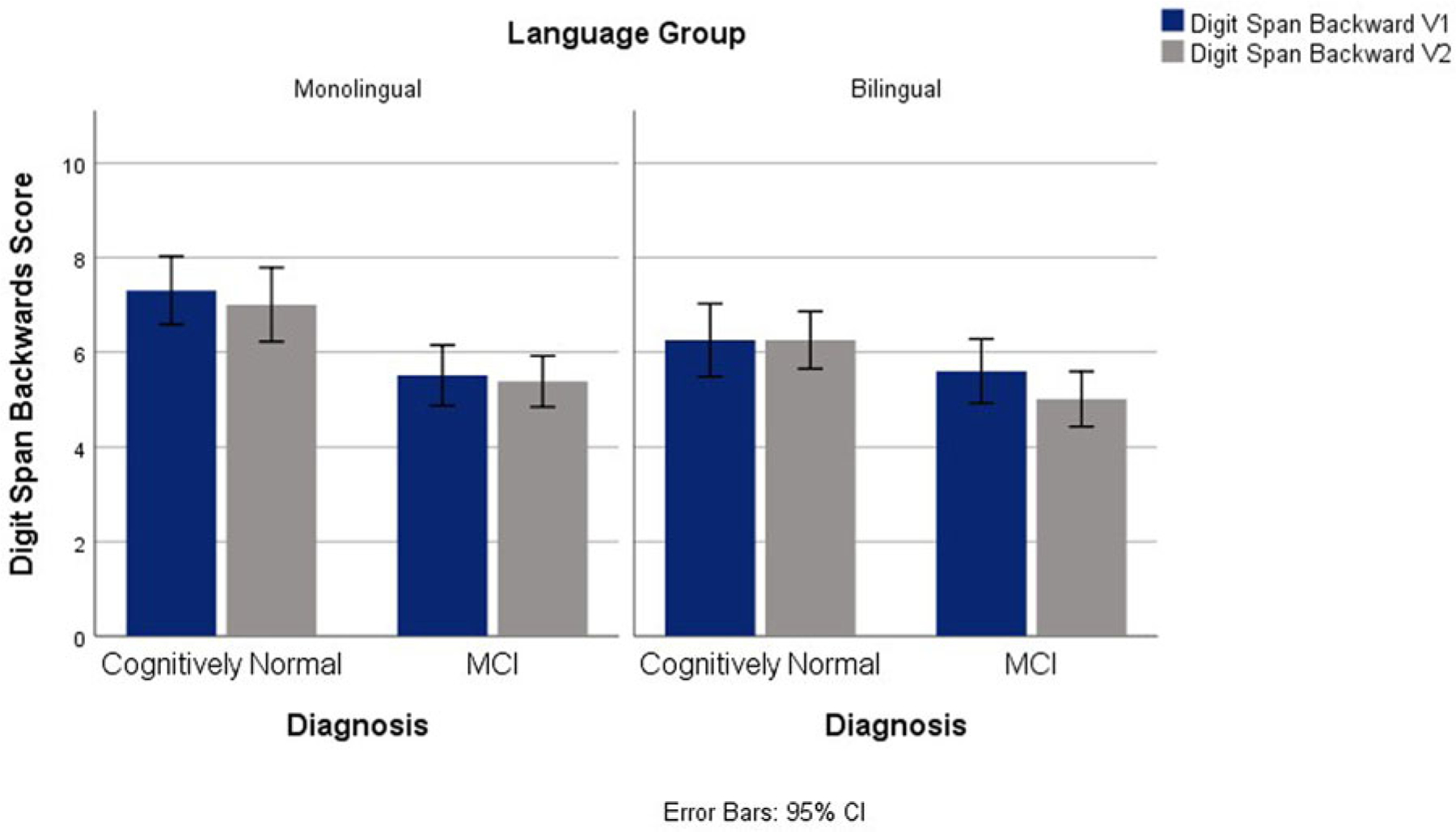
Digit Span Backwards Scores for V1 and V2 Across Diagnostic and Language Groups.

Significant main effects for language and diagnostic groups emerged for Digit Span Backwards between groups, *F*(1,145) = 18.99, *p* <.001, $\eta_{p}^{2}=.114$, and *F*(1,145) = 4.54, *p* = .035, $\eta_{p}^{2}=.030$, respectively. In general, the monolingual group outperformed the bilingual group and controls surpassed MCI. No significant interactions were found between language group and time or between language group and diagnosis. See Figure 1.

**Fig. 2.**
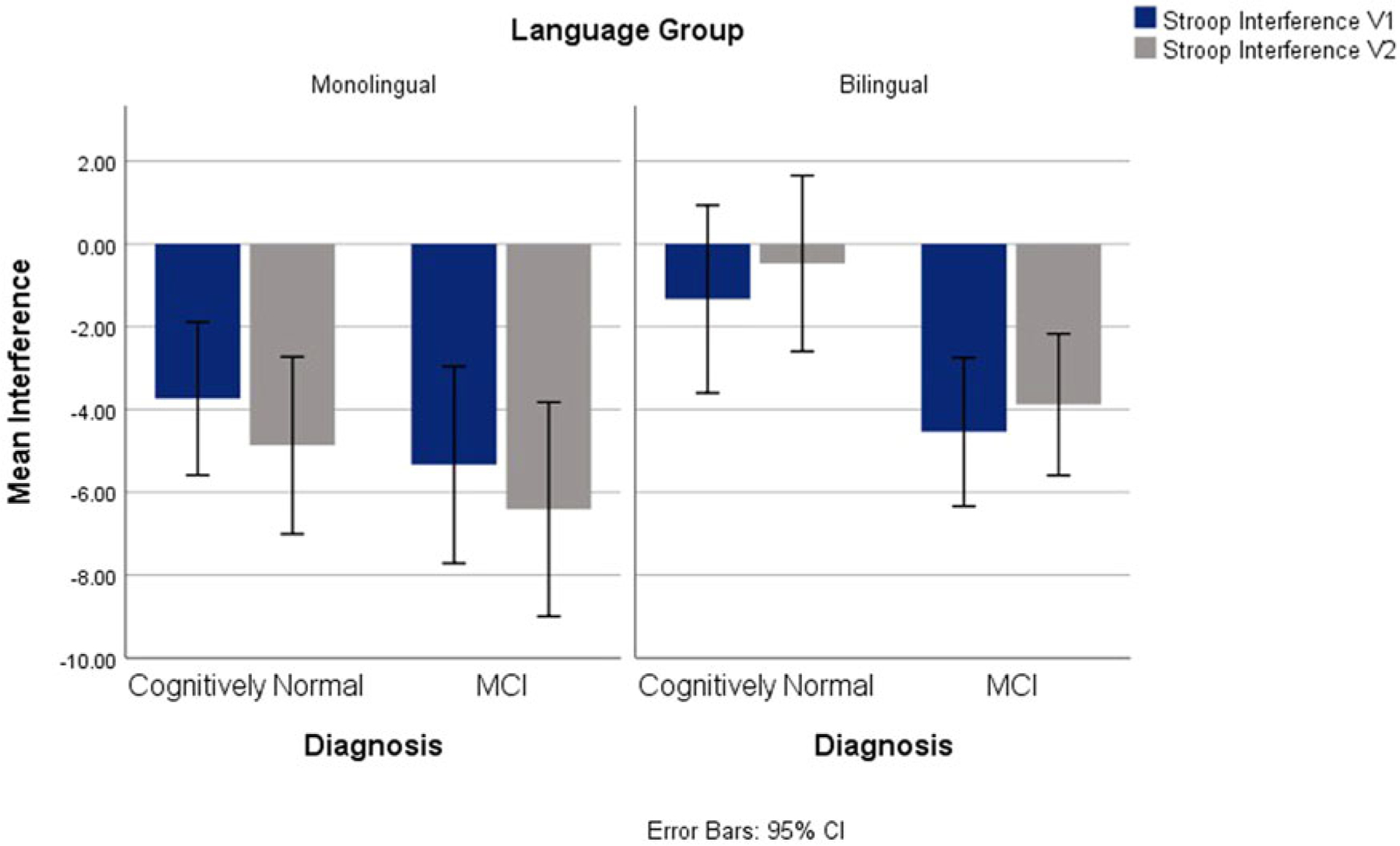
Stroop Interference for V1 and V2 Across Diagnostic and Language Groups.

On Stroop interference scores, main effects of language and diagnostic groups were observed, *F*(1,139) = 6.66, *p* = .011, $\eta_{p}^{2}=.046$, and *F*(1,139) = 6.47, *p* = .012, $\eta_{p}^{2}=.044$, correspondingly. Overall, the bilingual and the MCI groups had reduced Stroop interference compared to the monolinguals and control groups. No interactions were significant. See Figure 2.

**Fig. 3.**
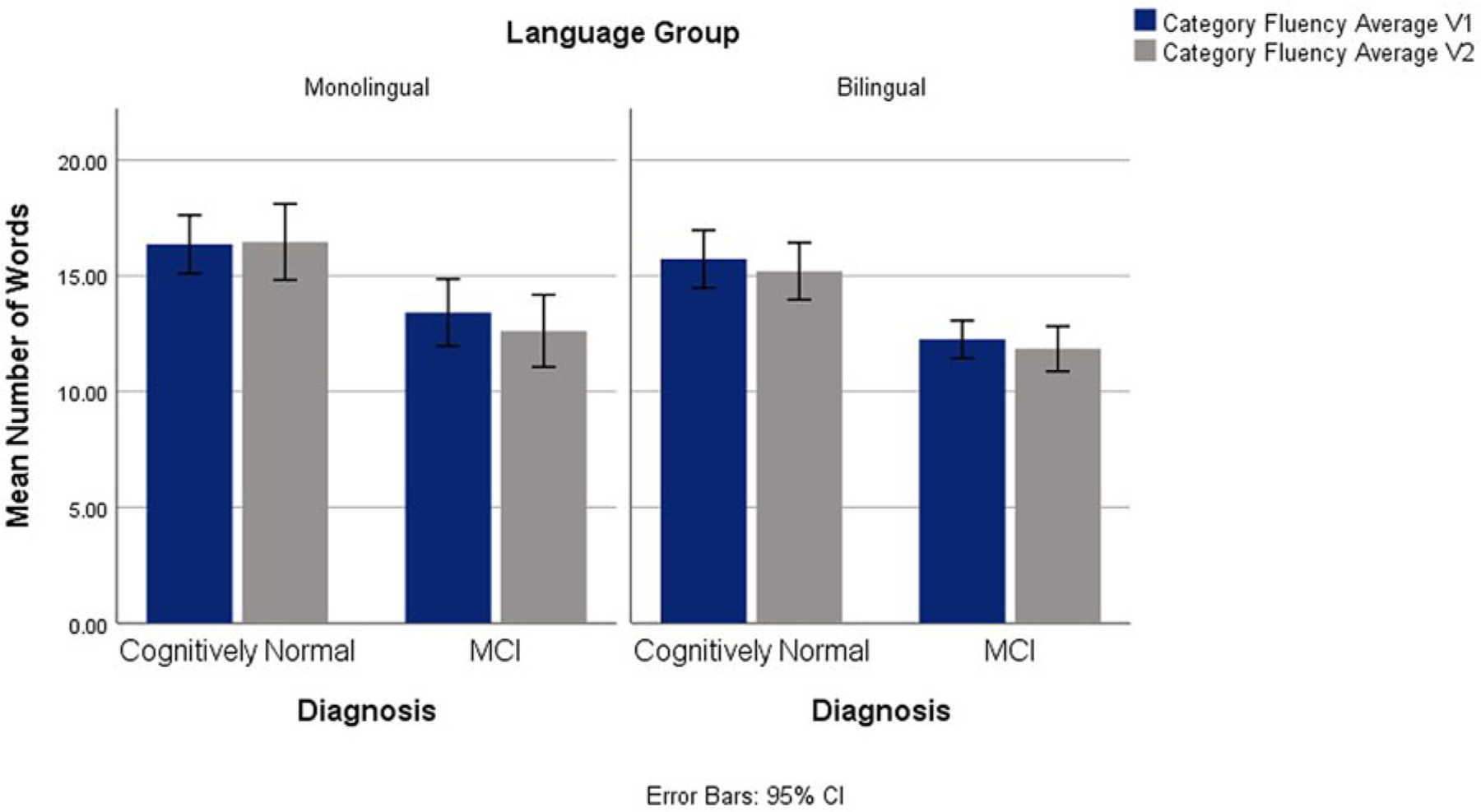
Category Fluency Average on V1 and V2 Across Diagnostic and Language Groups.

Main group effects were also found for category fluency average scores, *F*(1,127) = 4.36, *p* = .039, $\eta_{p}^{2}=.033$, with higher scores in the monolingual group and in the control group *F*(1,127) = 36.75, *p* < .001, $\eta_{p}^{2}=.222$. No interactions emerged. See Figure 3.

No significant language group effects or interactions were found for TMT-B minus TMT-A scores.

### Bilingualism characteristics

In addition to language group comparisons, the predictive value of the BI (assessing the degree of language balance) and the age of acquisition of English for CN or MCI participants who reported Spanish as their first language were examined.

#### Imaging

The initial analyses were performed with 115 Spanish–English bilinguals who had completed MRI scanning. This sample included CN, MCI, and dementia participants. Sex, diagnostic group, and variables were associated with bilingualism and therefore used as predictors, with the GMV of the left IFG and bilateral OFC used as dependent variables. The BI and English age of acquisition did not predict the GMV in these regions.

#### Executive function tests

These analyses were conducted with CN and MCI monolingual and bilingual participants (*n* = 111). The BI and English age of acquisition were used as predictors of V1 scores from Digit Span Backwards, Stroop interference, and category fluency average scores, as these measures exhibited language group differences.

There was a significant regression predicting category fluency average scores, *F*(5,105) = 14.68, *p* < .001, with the model predicting 38% of the variance as demonstrated by the adjusted R^2^. The BI was a significant predictor, *β* = −3.42, *t* = −2.30, *p* = .023, suggesting that this index, which reflects the balance in proficiency between languages, added significant predictive value to mean scores of category fluency over and above the other variables in the model.

A significant regression predicting Digit Span Backwards was also found, *F*(5, 103) = 3.64, *p* = .005, with the model predicting 11% of the variance. The age of acquisition of English was a significant predictor, *β* = −.06, *t* = −2.13, *p* = .035, adding significant predictive value to Digit Span Backwards scores over and above the other variables in the model.

Finally, the bilingualism variables did not predict Digit Span Backwards or Stroop interference.

### Relationship between decline and GMV

As previous research has reported delays in the onset of cognitive decline for bilinguals, the estimated age at which participants began exhibiting cognitive symptoms was also examined. One hundred and fifty-seven participants with MCI or dementia (64 monolinguals and 93 bilinguals) were included in this analysis. There were no language group differences in the estimated age of the onset of cognitive decline. Linear regression analyses were conducted to examine the influence of the left and right GMV of OFC, IFG, hippocampi, and entorhinal cortices over the estimated age of onset of cognitive decline. Education and language group were included as predictors. The model including the left and right OFC as predictors was significant, *F*(4,133) = 2.74, *p* = .03, with the model explaining 4.8% of the variance demonstrated by the adjusted R^2^. However, none of the individual predictors reached statistical significance. The model that included the left and right IFG was also significant, *F*(4, 133) = 4.44, *p* = .002, and explained 9.1% of the total variance (adjusted R^2^), with no predictors reaching statistical significance. The model including the left and right hippocampi as predictors was significant, *F*(4, 133) = 3.89, *p* = .005, and explained 7.7% of the variance (adjusted R^2^), with no predictors reaching significance. Lastly, the model that included the left and right entorhinal cortices was not statistically significant.

Results of the multiple linear regression indicated that the Stroop interference scores were significantly predicted by the collective effect between language group, age, education, sex, and left IFG GMV, *F*(5, 202) = 2.28, *p* < .05, R^2^ = .053. However, the only individual predictor that was marginally significant was education (*t* = 1.93, *p* = .05).

### Post-hoc analyses

Given that most evidence for cognitive reserve is reported in cognitively abnormal individuals (i.e., MCI and AD), the authors conducted post-hoc analyses in the MCI and dementia groups to examine whether there is a difference in the dissociation between GMV and task performance between monolingual and bilinguals. The monolingual and bilingual groups differed in age, *F*(1, 156) = 6.30, *p* = .013, therefore age was included as a covariate.

Univariate GLM comparing the groups in GMV of the selected regions revealed differences in the left and right IFG, *F*(1, 136) = 7.54, *p* = .007, and *F*(1, 136) = 3.94, *p* = .049. In both regions, bilinguals exhibited higher volume.

Additionally, univariate GLM were used to differences in task performance – namely, those in which language group differences were found in previous analyses (i.e., Stroop interference, Digit Span Backward, and Category Fluency). No significant differences were found.

Finally, to examine the association between GMV and task performance, three linear regression analyses were conducted using age, language group, diagnosis, and left and right IFG as predictors, and the Stroop interference, Digit Span Backward, and Category Fluency as dependent variables. The models predicting Stroop interference and Category fluency were not significant, while the model predicting Digit Span Backward was, *F* (5,132) = 3.96, *p* = .002, R^2^ = .13; diagnosis was the only significant predictor on the later model, *t* = −3.57, *p* < .001, suggesting that participants with a diagnosis of dementia have a higher chance than MCI participants to have low scores on Digit Span Backwards.

## Discussion

This study compared monolingual and bilingual participants with normal and abnormal aging in GMV of regions associated with memory, language, and EF. Bilinguals exhibited higher GMV in areas associated with language and EF; however, no volumetric differences were found in areas related to memory. There was no evidence of a longitudinal (over an average period of 14 months) bilingual advantage on EF performance across CN and MCI monolingual and bilingual participants. However, there were general language group differences on overall EF scores, with monolinguals outperforming bilinguals on Digit Span Backwards and category fluency average, and bilinguals exhibiting reduced Stroop interference compared to monolinguals. A greater degree of balanced bilingualism was associated with reduced category fluency scores and acquiring English at a younger age was related to better performance on Digit Span Backwards. No differences in age of cognitive decline or a relationship between increased GMV and EF performance were found. Overall, the influence of bilingualism in this sample did not appear to contribute to cognitive reserve. However, moderate EF advantages in bilinguals were found. These findings are further discussed in the following sections.

### Imaging

The bilingual group exhibited higher GMV in the left IFG and left and right OFC, but no differences were found in regions related to memory. It was expected that due to the increased demand for language control, bilinguals would exhibit higher GMV in frontal regions associated with language and executive control. Partially supporting the hypotheses, bilinguals had higher GMV in the left IFG independent of diagnosis. The left IFG, which corresponds to Broca’s area, is strongly associated with language, and previous studies have reported higher volume and thickness in this region in bilingual individuals (Heim et al., 2019; Klein, Mok, Chen & Watkins, 2014), and activation differences in this region are reported in bilinguals depending on language proficiency (Golestani et al., 2006). Moreover, late bilinguals could activate distinct regions within Broca’s area for native and second languages (Kim, Relkin, Lee & Hirsch, 1997). Recently, it has been suggested that a common neural network of activation subserving first and second languages exists and includes the inferior frontal cortex, the anterior cingulate cortex (ACC), the left basal ganglia, and the inferior parietal/supramarginal gyrus (Abutalebi & Green, 2007; García-Pentón et al., 2016), while other views indicate that some structures are only involved with a weaker second language (Mouthon, Annoni & Khateb, 2013). Therefore, the finding of increased GMV in the left IFG in bilinguals is congruent with previous research demonstrating an association between the active use of two languages and structural and functional brain plasticity. Previous findings suggest that age has a modulating effect on the bilingualism-brain relationship (Heim et al., 2019), with volumetric differences in the left IFG occurring earlier than in the IPL. While the present study did not examine the direct influence of age, this variable was significant in the analyses, suggesting that the neuroplastic effects of bilingualism vary across the lifespan. Furthermore, bilingualism predicted the GMV of the left IFG, with younger bilinguals more likely to exhibit higher GMV in this region.

In the left and right OFC, bilinguals exhibited higher GMV than monolinguals. However, this difference, influenced by clinical diagnosis, was only found in the dementia group.

The findings related to frontal language and EF regions partially support previous research suggesting that increased language experience and manipulation leads to neuroplastic changes (García-Pentón, Pérez Fernández, Iturria-Medina, Gillon-Dowens & Carreiras, 2014). Duncan et al. (2018) also described that the bilingual experience might act as “exercise” for regions involved in control processes, ultimately leading to changes reflected in the increased GM density.

Besides frontal language and EF regions, it was also expected that consistent with the theory of cognitive reserve, the bilingual group, matched in demographic variables with a monolingual group and controlling for diagnosis, would have reduced GMV in memory regions; this was not found in the present study. The current results differ from the findings of Duncan et al. (2018), which reported that bilinguals with AD had lower GMV in memory-related regions. It is important to note, however, that Duncan et al.’s sample was older than the present one, and the ROIs differed (these researchers selected the parahippocampal gyri and the rhinal sulci). Costumero et al. (2020) also reported lower volume in MCI bilinguals compared to monolinguals in regions related to brain atrophy in dementia. Besides ROI differences, our bilingual sample consisted of mostly immigrants, while Costumero et al.’s sample included native-born bilinguals. Lastly, Schweizer et al. (2012) analyzed computerized tomography scans for a sample with probable AD and described increased atrophy for bilinguals compared to monolinguals in areas related to AD. The discrepant results could be attributed to the evaluation of different age cohorts and the use of different neuroimaging techniques.

In general, the imaging data do support bilingualism as a factor for brain plasticity in specific areas of the aging brain. Although memory regions did not demonstrate language group differences, the larger volume in language regions (e.g., left IFG) could be attributed to the increased activation of these regions in bilinguals (Abutalebi & Green, 2007; García-Pentón et al., 2016). The left IFG is commonly associated with syntactic and morphological language processing; however, it is also related to increased cognitive control demands, such as those needed to successfully complete Stroop-type tasks (Chen, Lei, Ding, Li & Chen, 2013). The use of shared prefrontal neural circuitry during visual (color identification under Stroop conditions) and language tasks of cognitive control processes (sentence comprehension under conditions of syntactic ambiguity) have been reported (January, Trueswell & Thompson-Schill, 2009). Our results seem to suggest that bilingualism could improve inhibitory control during Stroop tasks and also increase the size of regions involved with inhibitory control (Abutalebi & Green, 2016). The behavioral (inhibitory control) and brain adaptations observed in the bilingual sample do not support the classical concept of cognitive reserve, but rather the concept of neural reserve appears to have influenced the findings. The structural brain adaptations observed in bilinguals may act as compensatory mechanisms for the increased demand for continuous efficient language control, providing the biological basis of the observed bilingualism-induced regional brain neuroplasticity (Pliatsikas, 2020) and observed as better performance in inhibitory control tasks (Bialystok, 2017; Salvatierra & Rosselli, 2011).

Differences between EF and memory regions and their associations with neuropsychological performance between the language groups were found; greater TMT-B minus TMT-A differences scores were associated with reduced GMV of the right hippocampi and left entorhinal cortex in bilinguals. Few studies have examined TMT and GMV relationships. Nestor et al. (2015) reported associations with faster TMT-B completion times and GMV of the left OFC and left middle orbital gyrus in healthy adults. In aging, TMT difference scores have been related to the volumes of the left ventrolateral prefrontal cortex, dorsolateral prefrontal cortex, and frontopolar cortex (Ruscheweyh et al., 2013). Differences between these results and the present ones might be related to different sample characteristics, with Ruscheweyh et al. excluding older adults with probable MCI, as well as different imaging methodology.

### Longitudinal Analyses

EF performance during V1 and V2 in CN and MCI monolingual and bilingual participants was analyzed. Despite nonsignificant longitudinal differences in scores between the language groups, differences were found on Digit Span Backwards and category fluency average scores, with monolinguals outperforming bilinguals on these tasks. The category fluency findings are consistent with previous research, which typically report lower scores on verbal tasks in bilinguals resulting from increased linguistic interference (Rosselli et al., 2000) and are in line with the hypothesis that monolinguals would have higher fluency scores than bilinguals. Performance on fluency tasks, particularly category fluency, is affected in AD (Weakley & Schmitter-Edgecombe, 2014). Findings from the current study contribute to the literature describing the disadvantages exhibited on verbal tasks in bilingual CN and MCI individuals.

Findings from the Digit Span Backwards did not agree with the prediction that, as a result of the increased demands of bilingualism on executive control, bilinguals would outperform monolinguals. Yang (2017) reported better performance on Digit Span tasks in an intermediate bilingual group (determined by language proficiency and use), while this finding was not replicated in a high bilingual group. Yang suggested that the intermediate bilingual group developed stronger WM abilities because of the WM demands of bilingualism, while the high bilingual group no longer experienced the demands of language monitoring and memorization.

As predicted, Stroop interference scores followed a different trend from the other tasks; bilinguals exhibited reduced interference compared to monolinguals. There is previous research suggesting an interference advantage in bilinguals (Bialystok et al., 2014b), while other authors report only a weak advantage (Donnelly, Brooks & Homer, 2019) or no advantage (Lehtonen et al., 2018). In the current study, it appears that the inhibitory requirements of bilingualism may not be confined to language control, but instead may share resources with domain-general EF processes (Bialystok, 2017; Green, 1998). Previous studies have suggested that bilingualism influences Stroop test scores (Rosselli, Ardila, Santisi, Arecco, Salvatierra, Conde & Lenis, 2002), and Suarez, Gollan, Heaton, Grant, Cherner, and HNRC Group (2014) reported that higher L2 fluency was associated with inhibitory advantages on the Stroop even when administered in the native language.

No language group differences in TMT difference scores were found. Research suggests a bilingual advantage on TMT (Suárez, 2013), while the opposite results are also reported (Kisser, Wendell, Spencer & Waldstein, 2012). In the Kisser et al. (2012) study, the sample included undergraduate students (with ages between 18–44 years), while the Suárez (2013) study included participants between the ages of 20–63. Furthermore, both studies used TMT-A and TMT-B separately as opposed to TMT difference scores, which is considered a better estimator of switching abilities (Kopp, 2011). Further research needs to examine the influence of aging in TMT completion times and related scores.

Because the analyses included only CN and MCI participants, changes in scores from V1 to V2 could be minimal and undetectable, and this could explain the present findings. As data collection is ongoing, it will be possible to explore whether considering data from subsequent visits can capture neuropsychological differences between the language groups. However, previous studies, such as Zahodne et al.’s (2014), did not find different rates of decline or dementia conversion between the language groups. Likewise, Mungas et al. (2018) did not find evidence of a bilingualism effect in cognitive decline.

### Bilingualism variables

Besides the language group comparisons, additional aims of this study involved the identification of factors within Spanish–English bilinguals that were predictive of GMV and EF performance differences. To this end, the influence of a BI (reflecting the language proficiency balance) and the age of English acquisition was explored. These variables did not predict GMV, and the BI only predicted category fluency scores, with greater linguistic balance increasing the probability of lower scores. It appears that higher proficiency balance is accompanied by increased interference, and this resulted in reduced category fluency scores. Rosselli, Ardila, Lalwani, and Vélez-Uribe (2016) reported that proficiency, more so than balance, was associated with EF advantages. The BI was expected to be a strong predictor of neuropsychological test performance and GMV. However, it appears that language proficiency balance was related only to the task with the strongest verbal component.

Likewise, the age of acquisition of L2 was not a significant predictor of GMV. However, a younger age of English acquisition was associated with better performance on the Digit Span Backwards task. Sörman et al. (2019) included L2 proficiency in their language group comparisons and did not report its significant influence on executive control systems in 50–75-year-old adults. An earlier age of L2 acquisition is associated with reduced processing costs in bilinguals, and language balance is also suggested to play a significant role in EF advantages (Soveri, Rodriguez-Fornells & Laine, 2011; Yow & Li, 2015). Findings from this study support a mild influence of L2 age of acquisition and linguistic balance. It is likely that if there are EF advantages to be found in bilinguals, they do not appear to stem strongly from these characteristics of the bilingual experience.

### Relationship between decline and GMV

The age at which cognitive decline began was not different between language groups. Additionally, the GMV of the selected regions was not a predictor of this variable, suggesting that, despite volumetric differences between the language groups, these do not influence the reported age of onset of cognitive decline in this sample. While this partly disagrees with several studies that report that bilinguals are older at the onset of dementia (Alladi et al., 2013; Woumans et al., 2015), these findings are in line with results from other researchers (Mungas et al., 2018; Zahodne et al., 2014) who used prospective assessments to establish this age. The present findings, like those reported by Alladi et al. and Woumans et al., used retrospective analyses for the age of symptom onset, while Mungas et al. and Zahodne et al. followed participants over time. Furthermore, the current sample included Hispanic American individuals, and therefore had a greater degree of cultural and linguistic similarity with participants from the Mungas et al. and Zahodne et al. studies compared to Alladi et al.’s participants from India and Woumans et al.’s European sample. Moreover, we found that the higher GMV in the left IFG was not associated with reduced Stroop interference.

Because cognitive reserve is often reported in MCI and AD (e.g., Berkes, Calvo, Anderson & Bialystok, 2021; Ramakrishnan, Mekala, Mamidipudi, Yareeda, Mridula, Bak, Alladi & Kaul, 2017), the relationship between GMV and task performance was additionally examined in these cohorts removing the control (i.e., cognitively normal) group. Similar to the findings from GMV and age of decline, we did not find a relationship between the increased GMV in the left and right IFG and task performance, and other GMV regions were similar between the language groups. This suggests that, in this sample, the effect of greater GMV in the IFG is not reflected in cognitive advantages for bilinguals with MCI or dementia, at least with the cognitive tests used in this study. However, results from the current study suggest that there is brain reserve in the frontal lobe in bilinguals with abnormal aging that is not seen in monolinguals with an equivalent diagnosis.

The absence of bilingual cognitive reserve in the current study is in contradiction to previous research (Berkes, Calvo, Anderson & Bialystok, 2021; Ramakrishnan et al., 2017). The discrepant results can be explained by methodological differences between studies. In the Berkes et al. study, bilingual participants were matched to monolingual participants on neural parameters derived from diffusion tensor imaging. Matched monolinguals had poorer clinical diagnoses than those predicted by chance from a theoretical null distribution, and poorer cognitive performances than matched bilinguals as measured by scores on the MMSE. In our study volumetric MRI was used as the biomarker predictor of several executive function tests instead of using a cognitive screening test (e.g., MMSE). Therefore, brain and cognitive parameters are not equivalent between the two studies. Future research should assess whether there is cognitive reserve in our sample using white matter integrity as a predictor. Ramakrishnan et al. (2017) found that bilingual MCI patients had a clinical onset of cognitive complaints 7.4 years later than monolinguals. As opposed to our participants, 64% of bilinguals spoke more than 2 languages (multilingualism) compared to our study in which all bilinguals spoke only two languages (bilingualism). Delays in cognitive decline have been associated with the number of spoken languages (Chertkow et al., 2010; Kavé, Eyal, Shorek & Cohen-Mansfield, 2008) and may explain the discrepancies.

### Study strengths, limitations, and future directions

Limitations of the current study include an overrepresentation of females and MCI participants. V2 data for the dementia group was limited; therefore, the longitudinal neuropsychological performance was not assessed in this group. Future studies should include a greater number of participants diagnosed with dementia. Additionally, it remains to be seen whether similar results would be obtained when different types of bilinguals are included (i.e., weak vs. strong bilinguals; Ardila, 2007).

Category fluency tasks, which require greater lexical than executive demands, often demonstrate a monolingual advantage due to cross-linguistic interference in bilinguals (Paap et al., 2017). Therefore, future studies should include other types of fluency tasks to overcome this limitation.

In the current study, the time between the two visits was highly variable. The 10–33-month interval likely influenced the longitudinal findings; however, the number of months between visits did not differ across our groups and was not associated with neuropsychological change scores. Zahodne et al.’s (2014) visit interval was 18–24 months, while Mungas et al.’s (2018) sample was evaluated every 12–15 months; therefore, in the present study, the broader range might partially explain the disparity of findings between these previous studies and the current one.

Our language groups were formed based on subjective self-assessments of language proficiency in speaking, understanding, and reading. Use of objective measures of linguistic abilities is likely more accurate in determining language proficiency. Several studies have used similar methods of classification (e.g., Mungas et al., 2018; Woumans et al., 2015); however, the use of interviews to ascertain language abilities in future research is essential. Additionally, while steps were taken to achieve a relatively homogenous bilingual sample (e.g., excluding participants who reported a second language besides English or Spanish), this group included individuals from 10 Spanish-speaking countries (excluding Puerto Rico), with the majority being immigrants. The influence of immigration continues to be a significant confound in bilingualism research (Fuller-Thomson & Kuh, 2014), and, when possible, future studies should replicate these findings with participants matched on immigration status. However, recent findings (Duncan et al., 2018) regarding equivalent volumetric effects of bilingualism in immigrant and non-immigrant bilinguals with MCI suggest that immigration may not be as significant a factor as previously believed. Similarly, the monolingual group included individuals from different cultural backgrounds. Future research should include a homogenous sample of monolingual participants. While the language groups did not differ in occupation and years of education, employment data were not available for all participants. Furthermore, income information was not available for comparison. Therefore, it is possible that the measures employed did not capture other socioeconomic differences, and results should be interpreted with caution.

Another limitation was the retrospective nature of assessing the age at which cognitive decline began, as well as the limited number of participants with this information available. Alladi et al. (2013) and Bialystok et al. (2007) used a similar methodology to establish the age of symptom onset. However, as noted in Mukadam et al. (2017), these types of assessments are more likely to be influenced by other factors (e.g., education).

It could be argued that using a 1.5 SD cut-off for MCI might present a less stringent classification of the MCI diagnosis compared to the actuarial diagnostic method proposed by Jak and Bondi (Bondi, Edmonds, Jak, Clark, Delano-Wood, McDonald, Nation, Libon, Au, Galasko & Salmon, 2014; Jak, Bondi, Delano-Wood, Wierenga, Corey-Bloom, Salmon & Delis, 2009), which requires impairment of 1.0 SD or below the mean on two or more measures within a cognitive domain and have been shown to be more stringent than the conventional criteria for MCI. We utilized the most widely used approach of clinical diagnosis of MCI using a stringent clinical interview, an informant, and the use of a formal CDR Global score of .5. Given that we had two primary, albeit sensitive, memory measures and only a handful of additional measures representing other cognitive domains (with adequate, education, gender, and language norms), we augmented this approach with additional neuropsychological criteria. The actuarial diagnostic method proposed by Jak and Bondi (Bondi et al., 2014; Jak et al., 2009) requires impairment of 1.0 SD or below the mean on two or more measures within a cognitive domain. This is reasonable if there are multiple measures per cognitive domain (e.g., memory). However, a 1.5 SD cut-off is much more appropriate when each domain is represented by a modest number of measures, and it is employed in the vast majority of studies in AD research (see Brooks & Loewenstein, 2010). FreeSurfer provides brain volumes for numerous regions in both the left and right hemispheres. Given the characteristics of our older sample, the study focused on specific brain regions (medial temporal and frontal) that have been previously associated with the neuropsychological measures employed. Nonetheless, additional brain regions and their connections are likely involved in bilingualism and may not be assessed using structural MRI. This limitation is worthy of further research. Moreover, arguments for a shift in reliance and brain plasticity towards subcortical regions with prolonged bilingual experience have been suggested (e.g., Grant et al., 2014; Pliatsikas, 2020); however, the current study did not include any of these representative brain regions (e.g., caudate nucleus, thalamus, and cerebellum). The current findings should be complemented by incorporating these regions in future research.

Despite these limitations, the strengths of this study included a large sample size (imaging sample *n* = 214 and longitudinal sample *n* = 153), the use of four tasks to assess the construct of EF, and the exclusion of individuals who were not US-born or Hispanics who did not have the shared experience of immigrating to the US. The bilingual sample included individuals with high Spanish and English proficiency; therefore, these results can be generalized to highly fluent Spanish–English bilinguals who are immigrants to the US and who have resided in the US for a large portion of their lives. Furthermore, the use of longitudinal analysis in bilingual research remains scarce. As the 1Florida ADRC study is ongoing, the cognitive and imaging trajectories of these samples will be addressed in future longitudinal studies.

## Conclusion

In general, bilingualism appears to be associated with higher GMV in frontal regions related to language and EF, supporting the brain plasticity effect of the use of two languages. Results did not fully support the theory that bilingualism increases cognitive reserve; bilinguals were not older at the onset of cognitive decline, and there was no evidence of reduced GMV in regions related to memory. EF scores over time were not influenced by bilingualism; however, the bilingual group exhibited overall reduced interference on the Stroop test, while the monolingual group had higher scores on Digit Span Backwards and category fluency. A more balanced bilingualism predicted lower category fluency scores, and a younger age of English acquisition was associated with better performance on Digit Span Backwards. Overall, it appears that bilingualism does not serve to increase cognitive reserve, offer protection against age-related changes in temporal regions associated with memory, or moderate the initial age of cognitive decline. However, bilinguals exhibited smaller Stroop interference costs and exhibited higher GMV in regions associated with language and executive function – notably the left IFG (with a trending similar effect in the right IFG). This pattern of results suggests that lifelong bilingualism may contribute to neural reserve in aging within this cohort.

## Figures and Tables

**Table 1.** Demographic Characteristics of the Imaging and Longitudinal Samples

Imaging Sample												
	Diagnosis											
	Cognitively Normal				MCI				Dementia			
	*M*		*B*		*M*		*B*		*M*		*B*	
	*M(SD)*	*n*	*M(SD)*	*n*	*M(SD)*	*n*	*M(SD)*	*n*	*M(SD)*	*n*	*M(SD)*	*n*
Age	71.38 (6.09)	34	69.37 (6.44)	41	72.55 (7.95)	42	70.91 (7.02)	64	75 (11.18)	14	70.11 (8.88)	19
Education	16.03 (3.66)	34	16.02 (2.74)	41	14.74 (3.41)	42	15.14 (3.28)	64	14.57 (3.65)	14	13.89 (4.59)	19
Ethnicity												
EA		29		4		32		3		11	-	
Hispanic		5		37		10		61		3		19
% Female	67.60%		80.50%		50%		54.70%		64.30%		68.40%	
Longitudinal Sample												
	Diagnosis											
	Cognitively Normal				MCI							
	*M*		*B*		*M*		*B*					
	*M(SD)*	*n*	*M(SD)*	*n*	*M(SD)*	*n*	*M(SD)*	*n*				
Age	70.93 (6.30)	30	69.97 (6.3)	36	72.36 (7.61)	33	70.81 (7.27)	54				
Education	15.83 (3.71)	30	15.72 (2.78)	36	14.48 (2.99)	33	15.07 (3.58)	54				
Ethnicity												
EA		24		3		26		4				
Hispanic		6		33		7		50				
Sex												
% Female	66.70%		80.60%		63.60%		53.70%					

*Note.* MCI = Mild Cognitive Impairment; M = Monolingual; B = Bilingual; EA = European American.

**Table 2.** Gray Matter Volume Across Diagnostic and Language Groups

	Diagnosis											
	CN				MCI				Dementia			
	M		B		M		B		M		B	
	*M*	*SD*	*M*	*SD*	*M*	*SD*	*M*	*SD*	*M*	*SD*	*M*	*SD*
Left Hipp.	0.0024	0.0003	0.0025	0.0003	0.0023	0.0004	0.0024	0.0003	0.0021	0.0003	0.0021	0.0003
Right Hipp.	0.0025	0.0003	0.0027	0.0003	0.0024	0.0003	0.0024	0.0003	0.0022	0.0003	0.0022	0.0003
Left Ento.	0.0012	0.0002	0.0013	0.0002	0.0012	0.0003	0.0012	0.0003	0.001	0.0003	0.001	0.0002
Right Ento.	0.0012	0.0002	0.0012	0.0002	0.0012	0.0003	0.0011	0.0003	0.0009	0.0003	0.001	0.0003
Left IFG	0.0062	0.0008	0.0064	0.0007	0.006	0.0007	0.0063	0.0009	0.0054	0.0011	0.0065	0.0007
Right IFG	0.0063	0.0009	0.0064	0.0007	0.0062	0.0007	0.0064	0.0008	0.0055	0.001	0.0063	0.001
Left OFC	0.0079	0.001	0.0082	0.0007	0.0078	0.0007	0.0078	0.0009	0.0066	0.0014	0.0078	0.0009
Right OFC	0.0079	0.0008	0.0082	0.0007	0.0079	0.0007	0.0078	0.0008	0.0066	0.0013	0.0079	0.0009

*Note.* CN = Cognitively normal; MCI = Mild Cognitive Impairment; M = Monolingual; B = Bilingual; Left = Left Hemisphere; Right = Right Hemisphere; Hipp = Hippocampus; Ento = Entorhinal cortex; IFG = Inferior frontal gyrus; OFC = Orbitofrontal cortex.

Values presented as a percentage of intracranial volume.

**Table 3.** Correlations Between Neuropsychological Scores and Gray Matter Volume of Bilateral Hippocampi and Entorhinal Cortices in Monolinguals

	DSB	Stroop	TMT	Fluency	Left Hipp.	Right Hipp.	Left Ento.	Right Ento.
DSB	-							
Stroop	0.178	-						
TMT	−.450**	−.307**	-					
Fluency	.391**	0.09	−.408**	-				
Left Hipp.	0.187	0.068	−0.109	.376**	-			
Right Hipp.	0.12	0.077	−0.051	.344**	.833**	-		
Left Ento.	0.173	0.074	0.025	.216*	.497**	.470**	-	
Right Ento.	0.04	0.14	−0.126	.237*	.393**	.459**	.564**	-

*Note*. DSB = Digit Span Backwards; Stroop = Stroop Interference; Fluency = Category fluency; TMT = Trail Making Test B minus Trail Making Test A; Left = Left Hemisphere; Right = Right Hemisphere; Hipp. = Hippocampus; Ento. = Entorhinal cortex.

** Correlation is significant at the 0.01 level (2-tailed).

* Correlation is significant at the 0.05 level (2-tailed).

**Table 4.** Correlations Between Neuropsychological Scores and Gray Matter Volume of Bilateral Inferior Frontal Gyri and Orbitofrontal Cortices in Monolinguals

	DSB	Stroop	TMT	Fluency	Left IFG	Right IFG	Left OFC	Right OFC
DSB	-							
Stroop	0.178	-						
TMT	−.450**	−.307**	-					
Fluency	.391**	0.09	−.408**	-				
Left IFG	.256*	0.075	−0.187	.332**	-			
Right IFG	0.143	0.053	−0.181	.208*	.738**	-		
Left OFC	0.109	0.128	−0.171	.395**	.668**	.669**	-	
Right OFC	0.15	0.138	−0.16	.342**	.709**	.662**	.865**	-

*Note*. DSB = Digit Span Backwards; Stroop = Stroop Interference; Fluency = Category fluency; TMT = Trail Making Test B minus Trail Making Test A; Left = Left Hemisphere; Right = Right Hemisphere; IFG = Inferior frontal gyrus; OFC = Orbitofrontal cortex.

** Correlation is significant at the 0.01 level (2-tailed).

* Correlation is significant at the 0.05 level (2-tailed).

**Table 5.** Correlations Between Neuropsychological Scores and Gray Matter Volume of Bilateral Hippocampi and Entorhinal Cortices in Bilinguals

	DSB	Stroop	TMT	Fluency	Left Hipp.	Right Hipp.	Left Ento.	Right Ento.
DSB	-							
Stroop	.199*	-						
TMT	−.370**	−.298**	-					
Fluency	.348**	0.178	−.504**	-				
Left Hipp.	0.055	−0.06	−.344**	.328**	-			
Right Hipp.	0.033	0.117	−.341**	.301**	.791**	-		
Left Ento.	0.141	−0.007	−.259**	.283**	.455**	.430**	-	
Right Ento.	0.025	0.124	−.236**	.264**	.344**	.472**	.673**	-

*Note.* DSB = Digit Span Backwards; Stroop = Stroop Interference; Fluency = Category fluency; TMT = Trail Making Test B minus Trail Making Test A; Left = Left Hemisphere; Right = Right Hemisphere; Hipp. = Hippocampus; Ento. = Entorhinal cortex.

* Correlation is significant at the 0.05 level (2-tailed).

** Correlation is significant at the 0.01 level (2-tailed).

**Table 6.** Correlations Between Neuropsychological Scores and Gray Matter Volume of Bilateral Inferior Frontal Gyri and Orbitofrontal Cortices in Bilinguals

	DSB	Stroop	TMT	Fluency	Left IFG	Right IFG	Left OFC	Right OFC
DSB	-							
Stroop	.199*	-						
TMT	−.370**	−.298**	-					
Fluency	.348**	0.178	−.504**	-				
Left IFG	0.114	−0.057	−0.082	−0.042	-			
Right IFG	0.023	−0.029	−0.097	0.028	.620**	-		
Left OFC	0.112	0.124	−.318**	.211*	.424**	.407**	-	
Right OFC	0.049	0.111	−.181*	0.01	.521**	.457**	.742**	-

*Note*. DSB = Digit Span Backwards; Stroop = Stroop Interference; Fluency = Category fluency; TMT = Trail Making Test B minus Trail Making Test A; L = Left Hemisphere; R = Right Hemisphere; IFG = Inferior frontal gyrus; OFC = Orbitofrontal cortex.

* Correlation is significant at the 0.05 level (2-tailed).

** Correlation is significant at the 0.01 level (2-tailed).

**Table 7.** Neuropsychological Scores During Visit 1 (V1) and Visit 2 (V2) Across Diagnostic and Language Groups

	Diagnosis			
	CN		MCI	
	M *M(SD)*	B *M(SD)*	M *M(SD)*	B *M(SD)*
DSB V1	7.30 (1.93)	6.25 (2.29)	5.50 (1.78)	5.59 (2.49)
DSB V2	7.00 (2.10)	6.25 (1.79)	5.33 (1.49)	5.00 (2.14)
TMT V1	51.83 (30.42)	56.19 (35.24)	90.81 (64.38)	99.15 (79.13)
TMT V2	62.27 (55.57)	55.94 (41.88)	98.62 (58.80)	88.51 (61.81)
Stroop V1	−3.74 (4.95)	−1.33 (6.70)	−5.25 (6.40)	−4.41 (6.15)
Stroop V2	−4.87 (5.73)	−0.47 (6.27)	−6.41 (7.05)	−4.16 (6.22)
Fluency V1	16 (3.24)	15.54 (3.21)	13.54 (3.57)	12.32 (2.85)
Fluency V2	16.46 (4.07)	15.19 (3.36)	12.62 (3.86)	11.84 (3.45)

*Note*. CN = Cognitively normal; MCI = Mild Cognitive Impairment; M = Monolingual; B = Bilingual; DSB = Digit Span Backwards; TMT = Trail Making Test B minus Trail Making Test A; Stroop = Stroop Interference; Fluency = Category fluency.
